# A simple method for generating long-term Holocene climate data with future climate projections from meteorological observation data

**DOI:** 10.1016/j.mex.2025.103265

**Published:** 2025-03-13

**Authors:** Jake Tuuli, Andy J. Baird, Dylan M. Young, Andrew Duncan, Roxane Andersen

**Affiliations:** a The Environmental Research Institue, North West and Hebrides, The University of the Highlands and Islands, Thurso, KW14 7JD, UK; b School of Geography, University of Leeds, Leeds, LS2 9JT, UK; c UHI Inverness, The University of the Highlands and Islands, Inverness, IV2 5NA, UK

**Keywords:** Paleoclimate reconstruction, Climate projection, Peatlands, Site-specific paleoclimate series generation with integrated climate projections

## Abstract

Peatlands play a crucial role in global carbon storage, yet their resilience to climate change remains uncertain. This study presents a novel method for generating long-term (>1000 years) site-specific climate data to drive peatland ecohydrological models. Using meteorological observations, we employ the Long Ashton Research Station Weather Generator (LARS-WG) to produce stochastic climate series for precipitation and temperature. The method integrates Holocene climate reconstructions from the EPOCH-2 database to simulate paleoclimate trends and interpolates climate projections based on Shared Socioeconomic Pathways (SSP) from CMIP6 models. Finally, a time series of potential evapotranspiration is calculated using a modified version of the Thornthwaite equation. This approach ensures continuity in climate inputs for peatland modelling, aiding in the assessment of long-term climate impacts on carbon dynamics. Our method provides a replicable framework for other regions, supporting improved climate-driven peatland simulations.

• Long-term paleoclimate data with climate projections tailored to specific sites are scarcely available
• This research outlines a simple method for generating climate series for driving ecosystem models
• Uses open-source resources and databases that are applicable across Europe

## Graphical abstract

**Figure:**
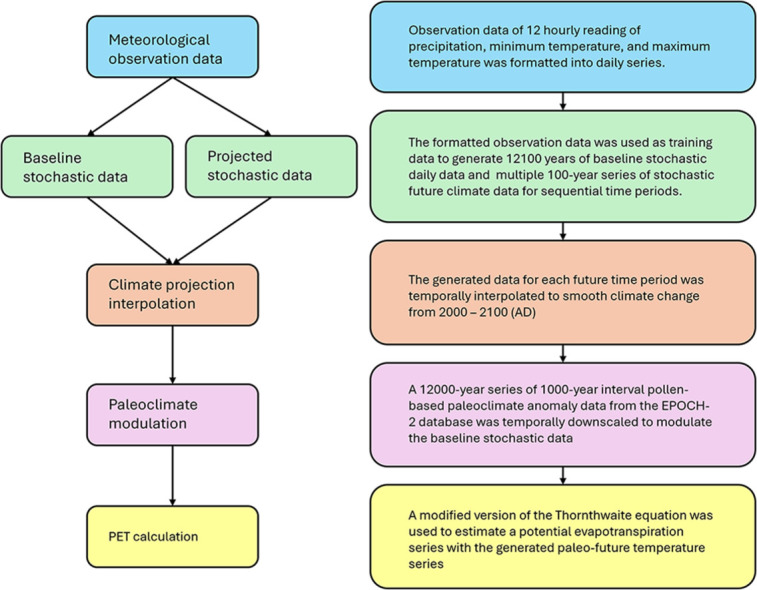

Specifications table

**Table:** 

Subject area:	Environmental Science
More specific subject area:	Climatology
Name of your method:	Site-specific paleoclimate series generation with integrated climate projections
Name and reference of original method:	
Resource availability:	UK Met observation data: https://catalogue.ceda.ac.uk/uuid/220a65615218d5c9cc9e4785a3234bd0/ LARS-WG: https://sites.google.com/view/lars-wg/ EPOCH-2: https://www.ncei.noaa.gov/access/paleo-search/study/18317

## Background

Peatlands are a globally significant ecosystem; they are estimated to cover 3% of the terrestrial surface area and store upwards of 600 Gt C [1]. Most of this carbon has been sequestered in the last 12,000 years. Peatlands may form where land becomes naturally saturated, and the addition of dead plant material is greater than its decomposition [2]. However, due to anthropogenic land use, peat-lands are releasing increased amounts of carbon as deeper water tables (caused by drainage) accelerate decomposition, in some cases shifting peatlands from a net carbon sink to a net source [3,4]. With rising global temperatures, there is concern for the resilience of peatlands and their ability to continue sequestering carbon as the metabolic activity driving decay increases with temperature [5]. However, peatlands have natural resilience mechanisms through eco-hydrological feedbacks that may help dampen the response to changing climatic condition conditions [6,7]. Peatlands are dynamic, complex systems, and modelling these landscape-scale processes is valuable for understanding their behaviour over decades to millennial time scales. Models aimed to simulate peatland ecohydrological processes require long-term (>1000 years) climate series as driving data, such as DigiBog [[8], [9], [10], [11], [12]], HPM [13], and MPEAT [14]. Furthermore, if simulating possible futures, continuous series of climate variables are required from a peatlands initiation to the end of the future projection. However, combined past and present site-specific climate data is not readily available and generating this data from scratch can be highly time-consuming. Here, we present a simple method for constructing weekly/monthly long-term (>1000 years) site-specific data series of precipitation, temperature, and potential evapotranspiration for driving data in a modern version of DigiBog. Furthermore, our method outlines how to generate a continuous series of climate data modulated with a paleoclimate reconstruction and future climate projection.

## Method details

This method aimed to create long-term (>1000 years) site-specific climate data at a sub-annual resolution (weekly or monthly) for use in a process-based peatland model. We wished to create the driving data for our model (DigiBog) to assess the impact of climate change on peatland development over the next 100 years using a range of climate projections. The climate input for the model requires a time series of precipitation, temperature, and evapotranspiration to simulate peat development in the past and future.

Here, we report the approach and resources used to create the climate series spanning 10,000 BCE to 2100 CE (i.e., 12,000 years in the past and 100 years in the future, given that 2000 CE is considered the present). The required inputs for DigiBog are relatively simple, meaning that the meteorological observation data required is often readily available as the driving data for our method. As a result, our method can be replicated across Europe. The minimum observation data required is at least one year of daily precipitation and minimum and maximum temperature data, site elevation, site coordinates, and the atmospheric CO2 concentration of the site. These data sets were used in the Long Ashton Research Station Weather Generator (LARS-WG) [15], which captures the seasonality and variability of the training data and generates a time series with similar statistical properties to be used as a baseline stochastic data series.

The weather generator was also used to create stochastic data for future climate scenarios from the same observation data, i.e., time series of 100 years of precipitation and temperature values modulated by the selected climate projection and time period. The method of interpolation between the baseline and future stochastic data sets is specified below.

Long-term climate anomaly data was used to modulate the baseline stochastic data for the paleoclimate reconstruction of air temperature and precipitation from 10000 BCE to 2000 CE. The EPOCH-2 database, used here, provides a Europe-wide 1000-year resolution series of climate anomaly values from a pollen-based reconstruction of the Holocene climate [16]. Continuous paleoclimate anomaly data extending back to 12000 BP is scarce and is often constrained to a single site. Other paleoclimate databases such as Temperature 12K [17] have been used in the reconstruction of Holocene temperature trends, such as in Erb et al. [18]; however, paleo reconstructions of precipitation are often missing. Finally, a potential evapotranspiration series was calculated using the Thornthwaite equation with the modulated stochastic climate data [19]. We decided to use the Thornthwaite equation over the widely used Penman-Monteith equation [20] as it requires variables such as irradiance and wind speed, which are scarcely available in paleoclimate records and climate projections.

### Meteorological observation data

We used observations from three meteorological stations from within the Flow country in the Highlands of Scotland, all located near peatlands, Table 1. Each site was chosen to represent a distinct part of the apparent precipitation gradient in the Scottish Highlands, spanning from the mountainous, Atlantic-facing region in the west, with annual precipitation upwards of 2000 mm, to the gently undulating areas to the east, which can experience as little as 600 mm of annual precipitation. Observation data for the three sites was accessed from The Met Office (2019): Met Office MIDAS Open: UK Land Surface Stations Data (1853-current) via the CEDA Archive [21]. The sites referred to here are: starting in the West, Cassley (CSS), Altnaharra (ALT), and Wick (WCK); see Table 1. The meteorological observations from these sites were extracted and processed as a continuous series of daily precipitation and minimum and maximum temperatures. All three sites had maintained observations for at least 20 years, suitably representing the sites’ climatology.

**Table 1:** Summary of climate and location data from three sites in the Highlands of Scotland.

Parameter	Altnaharra (ALT)	Cassley (CSS)	Wick (WCK)
Mean annual precipitation (mm)	1192.3	1965.6	774.0
Mean annual temperature (°C)	8.5	8.75	8.55
Latitude	58.288	58.168	58.454
Longitude	-4.442	-4.727	-3.09
Elevation (m)	81	99	36

### Stochastic weather generation

A stochastic weather generator was used to produce the baseline daily precipitation and temperature series for each site. The LARS-WG [15,22,23] is a lightweight and computationally efficient tool for generating representative stochastic time series of specific sites. Other weather generators, such as WGEN [24], AWEGEN [25], and CLIGEN [26] are also capable of generating the baseline data required for this method; however, LARS-WG was deemed to be the best combination of ease of use, computational demand, and site specificity alongside its climate projection capabilities. LARS-WG has featured in multiple weather generator comparison studies and has consistently performed well. Chen et al. [27] compared five weather (including LARS-WG, WGEN, and CLIGEN) generators by simulating daily precipitation and temperature for the Loess Plateau of China, where they concluded that LARS-WG consistently outperformed the other models in simulating the distribution of daily precipitation. Furthermore, Khazaei [28] evaluated the performance of four weather generators for simulating historical periods and downscaling future climate projections. Khazaei [28] concluded that LARS-WG, alongside the other weather generators evaluated, performed well at reproducing historical series and the monthly means of temperature and precipitation for future periods.

The specific version of the LARS-WG used here was LARS-WG 8.0. This version incorporates climate projections from six general circulation models from the most recent phase of the Coupled Model Intercomparison Project (CMIP6): ACCESS-ESM1-5 [29], CNRM-CM6-1 [30], GFDL-ESM4 [31], HadGEM3-GC31-LL [32], MPI-ESM1.2 [33], MRI-ESM2.0 [34]. These climate projections are based on the Shared Socioeconomic Pathways (SSP) from CMIP6: SSP1-2.6, SSP2-4.5, and SSP5-8.5. The SSPs are defined according to their projected increase in radiative forcing. A complete method of using LARS-WG comes with its installation. Additionally, detailed descriptions of LARS-WG are given in [35] and [36]. In short, LARS-WG uses daily observation data to generate semi-empirical probability distributions to approximate wet and dry day occurrence, and the amount of precipitation on a wet day is approximated by a semi-empirical distribution for a given month. Minimum and maximum daily temperatures are then modelled using two normal distributions depending on whether it is a wet or dry day. Daily means of temperature vary according to a third-order finite Fourier series.

We used LARS-WG to generate 12100 years of baseline climate data and 100 years of climate data for each climate projection. When selecting a climate projection, a time period must also be selected. The time periods increment every decade, from 2021-2040 to 2081-2100. We generated a dataset for each SSP and time period (for interpolation) available for the HadGEM3-ES GCM here as an example, totaling 22 data series (baseline + 3 SSPs x 7 time periods). Each daily series generated should then be reformatted to a sub-annual interval series for temperature and precipitation. This was done by first calculating the average of the generated minimum and maximum temperature series for each day as an approximation for the mean daily temperature. Then, the daily temperature and precipitation series we aggregated into either months or weeks. Precipitation was summed to calculate its sub-annual interval values, whereas temperature was averaged.

### Climate interpolation

We interpolated our climate projection data to smoothly transition from the baseline climate to the climate forecasted for 2100. As previously mentioned, the generated stochastic series, baseline and future, are steady-state series; there is no transition between the two. Therefore, some interpolation between the series is needed to move from the current climate to the future one.

A set of scaler parameters was calculated for every time period. The baseline series contains 12100 years of sub-annual interval data, and each future period series contains 100 years of sub-annual interval data. All of the data series were averaged into a single seasonal series where each sub-annual interval is an average of all values in a given series’ in that interval, i.e. all January precipitation and temperature values were averaged.

(1) $${\bar{X}}_{SAI}=\;\frac{1}{Y}\sum_{y=1}^{Y}X_{SAI,\;y}$$

Where *X_SAI_*^¯^ is a dummy variable representing the mean temperature or precipitation of a sub-annual interval (SAI) and Y represents the number of years in the data series. With each of the future seasonal series, a scaling parameter is calculated relative to the baseline seasonal series. This is done by taking the absolute difference between the baseline and future series for temperature.

(2) $$\Delta T_{SAI,\;future}=\;{\bar{T}}_{SAI,\;future}-\;{\bar{T}}_{SAI,\;base}$$

Unlike temperature, the scaler parameters for precipitation use the relative difference between baseline and future sub-annual interval averages.

(3) $$\Delta P_{SAI,\;future,\;rel}=\frac{{\bar{P}}_{SAI,future}-{\bar{P}}_{SAI,base}}{{\bar{P}}_{SAI,base}}$$

Now, each set of scaler parameters contains a single scaler value for each sub-annual time interval, 52 values for weekly or 12 for monthly series. Using the scaler parameter sets, a temporal interpolation was performed between time periods for each sub-annual interval, resulting in a 100-year series for each sub-annual interval (month or week) of a climate change scenario. The main benefit of this method is that the interpolation maintains how seasonality is projected to be affected in future scenarios. The temporal interpolation between scaler values uses a univariate spline from the SciPy module [37] in Python to increase the resolution of the scaler sets to that of the sub-annual interval. With these downscaled scaler series, the tailing 100 years of the baseline stochastic series can be modulated elementwise with its corresponding sub-annual interval series. This was done for each of the SSP scenarios, resulting in a 12100-year series where the final 100 years now represent a future climate scenario.

### Holocene climate reconstruction

We used a Holocene climate reconstruction from EPOCH-2 [16] to modulate the historical part of the baseline series. The pollen-based paleoclimate reconstruction extends as far back as 12,000 years and provides spatially-interpolated gridded series of anomaly data for temperature, precipitation, growing days above 5°C, and moisture balance. The data was formatted in 1° latitudinal and longitudinal grids at 1000-year intervals. As outlined in Mauri et al. [16], the climate anomaly results from the pollen reconstructions were spatially and temporally interpolated to provide a complete database across Europe. Due to their proximity, Cassley and Altnaharra fell within the same grid square, and Wick in another, resulting in the Holocene climates for Cassley and Altnaharra being the same. We used a univariate spline interpolation to temporally downscale the paleoclimate anomaly data to the resolution of the baseline simulated data with the same SciPy module as above. Interpolating this way provides a smoother series than a linear interpolation. The baseline simulated data was then modulated with the interpolated anomaly data with a piecewise addition of the two time series (for precipitation and temperature).

### Potential evapotranspiration

The final step was to create a series of evapotranspiration at the same length and intervals as the mean temperature and precipitation series. The Thornthwaithe equation [19] was chosen to calculate the potential evapotranspiration (PET) here, as it only requires information that is available from most observation sites. The equation was initially intended for calculating monthly values of PET; however, by including scaling parameters, the equation can be adjusted to calculate PET on any sub-annual time scale.

The three main steps are:

First, the annual heat index is calculated by summing the sub-annual interval heat indexes and then adjusting the interval as if it were months.

(4) $$I=\;\sum_{n=1}^{N}{\left(\frac{t_{n}}{5}\right)}^{1.514}\cdot \left(\frac{12}{N}\right)$$

Where *t_n_* is the mean air temperature of the sub-annual interval and N is the number of sub-annual intervals per year. Next, a value for PET is calculated for every interval of the stochastic series with Eq 5.

This equation calculates the PET assuming a 30-day interval at 12 hours per day.

(5) $$PET_{n}=16\cdot {\left(\frac{10\cdot t_{n}}{I}\right)}^{a}$$

where *a* is given by:

(6) $$a=\;\left(675\;\times 10^{-9}\cdot I^{3}\right)-\left(771\;\times 10^{-7}\cdot I^{2}\right)+\left(1792\;\times 10^{-5}\cdot I\right)+0.49239$$

Finally, the daylight hours for each interval in the year were calculated using the site latitude and middle Julian date for the sub-annual interval to adjust the initial calculation of *PET_n_*.

(7) $$L_{n}=\left(\frac{24}{\pi }\right)\cdot \;\cos^{-1}\left(-\tan\left(lat\right)\cdot \tan\left(\delta_{n}\right)\right)\;$$

Where *lat* is the latitude of the site and *δ_n_* is the solar declination angle during the sub-annual interval, which is given by:

(8) $$\delta_{n}=23.45\cdot \sin\left(\left(J_{n}-81\right)\cdot \frac{360}{365}\right)$$

where *J_n_* is the median Julian date of the sub-annual interval. A scaled version of *PET_n_* is given by:

(9) $$PET_{n}^{\prime }=PET_{n}\cdot \frac{L_{n}}{12}\cdot \frac{D_{n}}{30}$$

Where *D_n_* is the number of days in the sub-annual interval.

## Method validation

LARS-WG performed well when generating the baseline site-specific data. Since the simulated data is a representation of the stochastic nature of the observation data rather than a replication of it, comparing specific elements of the time series would be meaningless. However, the main point of comparison is how well the baseline simulated data captures the site's annual seasonality of temperature and precipitation.

**Fig. 1:**
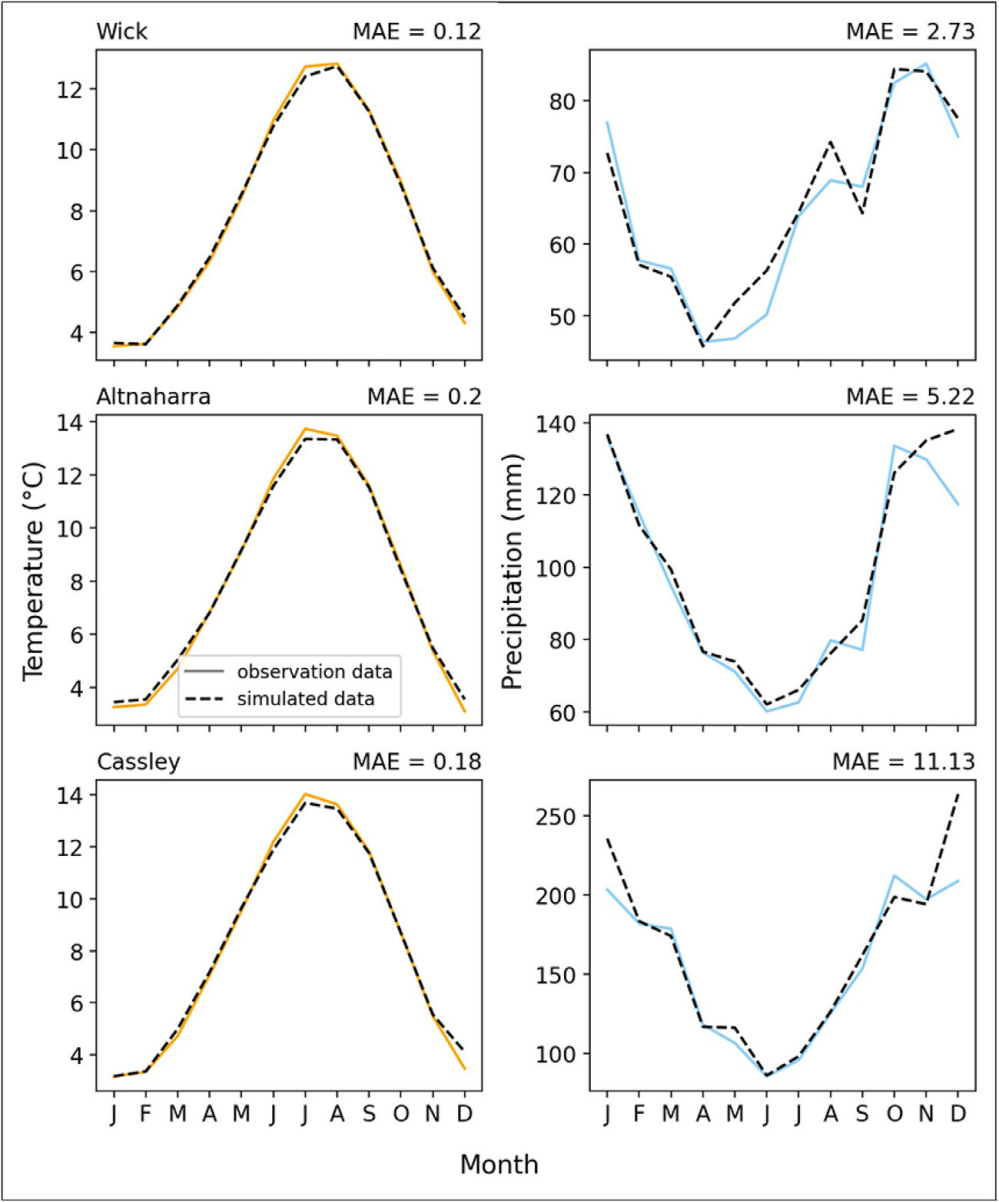
Shows the seasonal profile of temperature and precipitation for the three sites. The data was plotted in monthly intervals, and the mean absolute error (MAE) can be seen in its respective profile.

Fig. 1 shows that temperature seasonality is well captured by the simulated data for all three sites. Altnaharra had the largest mean absolute error (MAE) at 0.2 °C. July was consistently the most underestimated month when comparing the seasonal profiles, whereas December was consistently overestimated across all sites. As these are the extreme months for temperature, the more significant error could result from the stochastic data, when averaged, smoothing the extreme values. However, even the differences in the extreme months are minimal, averaging 0.35 °C for July and 0.43 °C for December for the three sites. Furthermore, we can see from Table 2 that any bias in the seasonal temperature values is very low suggesting that LARS-WG simulated the seasonality of tempereature well.

**Table 2:** A table of normalised seasonal bias for temperature and precipitation comparing the observed and baseline simulated data from LARS-WG for Altnaharra (ALT), Cassley (CSS), and Wick (WCK).

Bias	ALT	CSS	WCK
Temperature	0.34%	0.37%	-0.03%
Precipitation	2.9%	4.4%	1.3%

Unlike the temperature profiles, the observed seasonality of precipitation varied considerably between sites. Considering the shape of the seasonality profile (not the absolute values), the observed climates of Altnaharra and Cassley resemble each other more closely than Wick. The proximity of the Altnaharra and Cassley observation sites likely explains this. However, all seasonal distributions follow the same general pattern of higher precipitation rates at the start of the year, decreasing to a minimum and then increasing again in the winter months. The most notable difference between the profiles of Altnaharra and Cassley and Wick's profile is that the seasonal precipitation distribution is shifted by about two months earlier in the year. On average, the global minimum for precipitation at Wick occurs in May as opposed to June for the other sites. The simulated data captures the general inverted bell curve shape for all three sites; however, from Table 2 which shows the normalised bias for the three sites, we can see that all of the sites have a positive bias suggesting that precipitation is being overestimated. The overestimation is most clearly seen in December and January for Cassley and in December for Altnaharra. No adjustments were made to the data to account for this, although revised methods may wish to consider this.

### Climate projection analysis

**Fig. 2:**
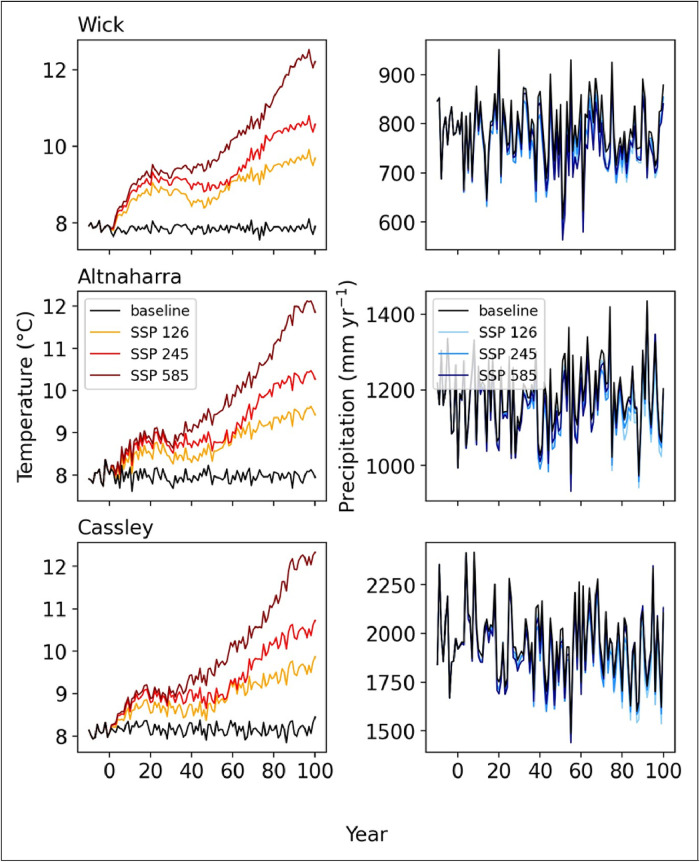
Comparison between climate projection scenarios for each site's stochastic temperature and precipitation series. The data here spans from year -10 (1990) to year 100 (CE 2100), while the climate projection starts at year 0.

As described, three future climate scenarios were generated for each site: SSP1-2.6, SSP2-4.5, and SSP5-8.5; these scenarios represent an increase of 2.6, 4.5, and 8.5 W m−2, respectively. For each site, temperature behaves as expected; scenarios with higher radiative forcing experience greater increases in annual average temperature after 100 years. In the projections from the GCM used here, temperatures increase significantly, peaking in CE 2090-2100 with warming greater than 4°C when considering SSP5-8.5 2. The three sites, on average, experienced 1.54 °C warming for SSP1-2.6, 2.4 °C warming for SSP2-4.5, and 4.01 °C warming for SSP5-8.5 after 100 years. In contrast, the precipitation profiles in Fig. 2 show how little the annual precipitation is predicted to change in these locations due to climate forcing relative to the variability between yearly precipitation rates. Considering the change in total precipitation over the 100 years, all three locations are forecast to receive less precipitation than the baseline, indicating the climate is becoming drier. The projections for Wick had the greatest relative reduction in precipitation, averaging 3.6% less precipitation per year when compared to the baseline climate.

### Holocene reconstruction

**Fig. 3:**
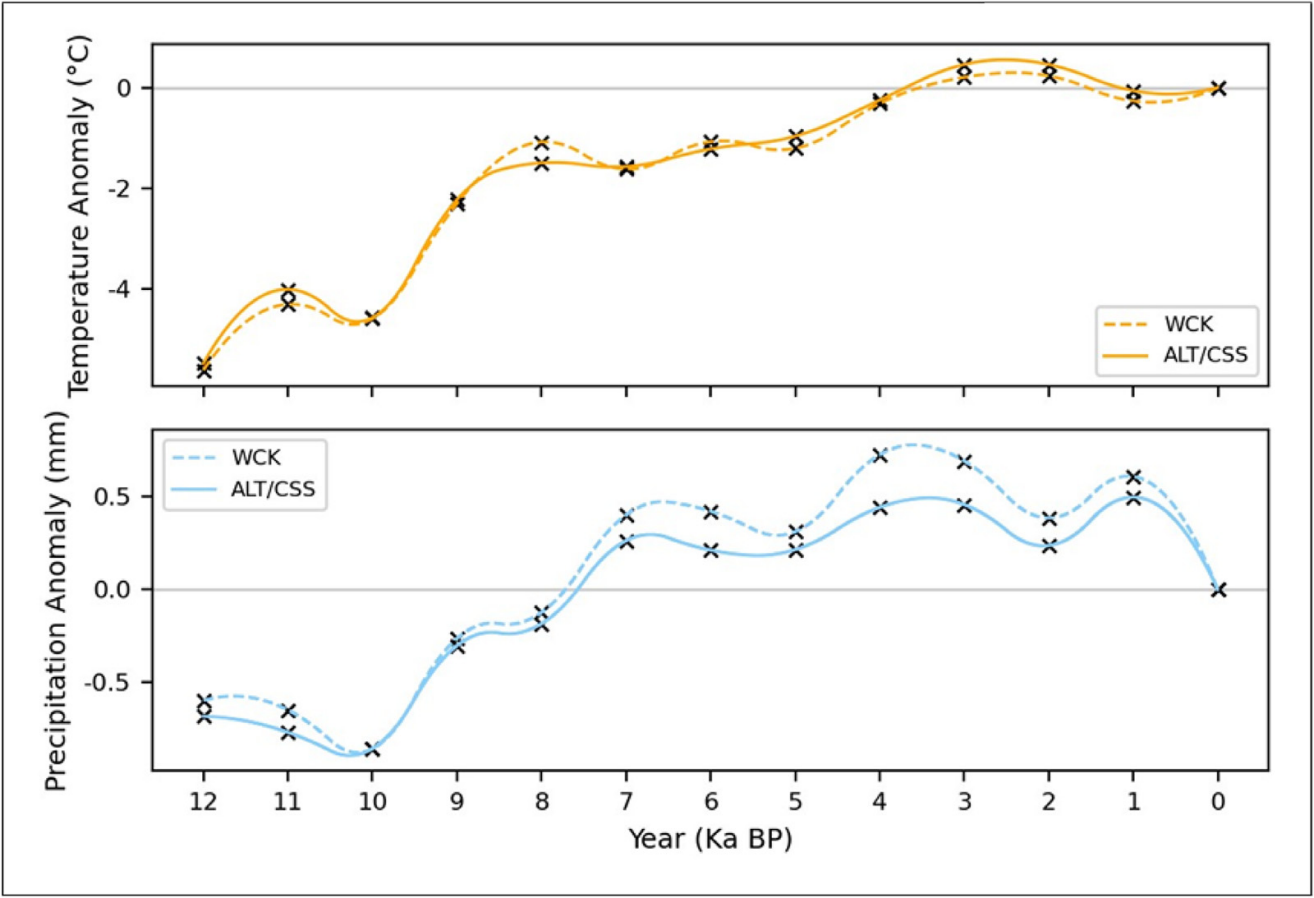
Shows daily anomaly data for temperature and precipitation over the last 12,000 years (the Holocene) interpolated from 1000-year anomaly data (marker = x) from a pollen-based reconstruction from the EPOCH-2 database. This data is location-specific for Altnaharra (ALT), Cassley (CSS), and Wick (WCK). Altnaharra and Cassley share the same anomaly data due to falling within the same grid square.

The Holocene reconstruction was based on the anomaly data available from the EPOCH-2 database. The anomaly data is in a gridded format, and because of this, Altnaharra and Cassley have the same anomaly profile, while Wick fell in the neighbouring grid square. Fig. 3 shows the anomaly data for the three sites alongside the interpolation used to smooth the data. For temperature, the climate warms quickly from 12-8 ka BP, rising about 4°C in 4000 years, where warming slows and reaches a maximum temperature anomaly of about 0.2 °C at 3-2 ka BP. As with temperature, the two daily precipitation anomaly series follow similar trends. After the minimum precipitation at 10 ka BP, precipitation rises from -0.9 mm day−1 to a wetter period starting around 7.5 ka BP. This wetter period lasts about 6000 years until 1 ka BP, where precipitation rates fall to today's climate. we can see that Wick, during the wet period of the Holocene (7.5-1 Ka BP), had a greater positive precipitation anomaly compared to Altnaharra/Cassley, suggesting Wick was relatively wetter from 7 ka BP to 0 ka BP (Fig. 3). For example, at the peak of precipitation for Wick (3.5 Ka BP), the region would have received an additional 285.1 mm of precipitation. In contrast, Altnaharra/Cassley would have received an additional 177.4 mm of precipitation during the same period.

### Potential evapotranspiration

Potential evapotranspiration calculated using the Thornthwaithe equation shows that, on average, the sites would be experiencing ∼ 597 mm per year from the baseline simulated data. This annual average sits within the range of PET for the region 400-700 mm [38], although it is on the higher end of the range. As the climate warms with increased radiative forcing in the SSP scenarios, PET increases with temperature as expected. In the most extreme radiative forcing scenario, SSP5-8.5, PET is projected to increase by 14.3 % for Wick and 13.3 % for Altnaharra and Cassley.

### Final paleoclimate series

**Fig. 4:**
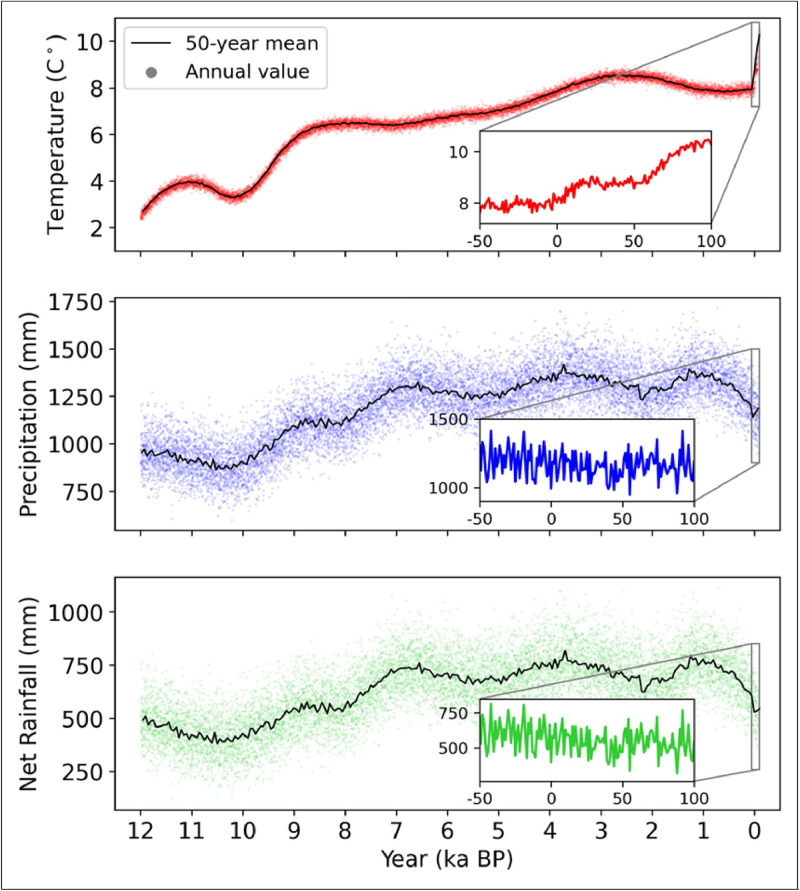
Shows an example of the final profiles for temperature, precipitation, and net rainfall (precipitation - potential evapotranspiration). Annual values are displayed with markers, while the black line plots the 100-year mean of the annual values. The climate here is simulated observation data from Altnaharra and projected with the SSP2-4.5 from HadGEM3-GC31-LL.

The final profiles for the paleoclimate temperature series show clear climate change during the Holocene, such as the rapid warming from 12 ka BP to 8.5 ka BP following the Last Glacial Maximum (LGM). We can see from Fig. 4a that the annual mean temperature series has a tight variance around the 50-year running mean. As a result, the temperature series is strongly influenced by the modulation of the Holocene anomaly data. For precipitation, the annual mean of precipitation varies much more around the 50-year running average; however, there are still long-term climate change trends present in Fig. 4b, on average, a gradual increase in annual precipitation until a maximum around 1 ka BP. Furthermore, Fig. 4c suggests that the annual potential evapotranspiration is tightly coupled with the annual average temperature as the profile exhibits the same trends throughout the Holocene.

## Limitations

This method benefits from multi-year data sets, with the weather generator documentation recommending greater than 10 years of daily climate data as the driving data for the stochastic series generation. Although the resources used here, LARS-WG and EPOCH-2, have both been validated across Europe, we have only tested this method on sites in oceanic/hyperoceanic climates. The use of this method outside of this range should be done with consideration of this. The temperature-based approach for calculating evapotranspiration may be especially sensitive to different climates which may lead to biases; however, because of the limitations on data availability, it remains a suitable choice here. Although other methods, such as the Penman-Monteith equation, may provide more accurate estimations of evapotranspiration, they would not be compatible with the temporal extent and resolution of this method. The method of temporal downscaling used here, univariate spline interpolation, while suitable, could potentially be improved. One iteration on this method could be to collapse the future and past temporal downscaling into a single interpolated series, guaranteeing a smooth transition between paleo and future series.

## Ethics statements

Not applicable.

## CRediT author statement

**Jake Tuuli:** Methodology, Analysis, Visualisation, Writing – original draft, Writing – review & editing. **Andy J. Baird:** Writing – review & editing. **Dylan M. Young:** Writing– review & editing. **Andrew Duncan:** Writing– review & editing. **Roxane Andersen:** Writing– review & editing.

## Declaration of competing interest

The authors declare that they have no known competing financial interests or personal relationships that could have appeared to influence the work reported in this paper.

## Data Availability

Data will be made available on request.
